# Role of Ubiquilins for Brown Adipocyte Proteostasis and Thermogenesis

**DOI:** 10.3389/fendo.2021.739021

**Published:** 2021-09-28

**Authors:** Carolin Muley, Stefan Kotschi, Alexander Bartelt

**Affiliations:** ^1^ Institute for Cardiovascular Prevention (IPEK), Ludwig-Maximilians-University, Munich, Germany; ^2^ German Center for Cardiovascular Research, Partner Site Munich Heart Alliance, Technische Universität München, Munich, Germany; ^3^ Institute for Diabetes and Cancer (IDC), Helmholtz Center Munich, German Research Center for Environmental Health, Neuherberg, Germany; ^4^ Department of Molecular Metabolism & Sabri Ülker Center, Harvard T.H. Chan School of Public Health, Boston, MA, United States

**Keywords:** ubiquilins, brown adipose tissue, cold adaptation, thermogenesis, proteostasis, ubiquitin-proteasome-system

## Abstract

The acclimatization of brown adipose tissue (BAT) to sustained cold exposure requires an adaptive increase in proteasomal protein quality control. Ubiquilins represent a recently identified family of shuttle proteins with versatile functions in protein degradation, such as facilitating substrate targeting and proteasomal degradation. However, whether ubiquilins participate in brown adipocyte function has not been investigated so far. Here, we determine the role of ubiquilins for proteostasis and non-shivering thermogenesis in brown adipocytes. We found that *Ubqln1, 2* and *4* are highly expressed in BAT and their expression was induced by cold and proteasomal inhibition. Surprisingly, silencing of ubiquilin gene expression (one or multiple in combinations) did not lead to aggravated ER stress or inflammation. Moreover, ubiquitin level and proteasomal activity under basal conditions were not impacted by loss of ubiquilins. Also, non-shivering thermogenesis measured by norepinephrine-induced respiration remained intact after loss of ubiquilins. In conclusion, ubiquilin proteins are highly abundant in BAT and regulated by cold, but they are dispensable for brown adipocyte proteostasis and thermogenesis.

## Introduction

Brown adipose tissue (BAT) is a unique organ that transforms chemical energy from nutrients into heat in response to cold, a process called non-shivering thermogenesis (NST) (1). While acute cold exposure activates the existing thermogenic potential, sustained cold induces the recruitment of thermogenic capacity. This process of cold adaptation requires tremendous remodeling of the cells and tissue, which involves both differentiation of new thermogenic adipocytes and increasing metabolic activity of the existing ones (1). Paradoxically, despite the catabolic nature of NST, long-term adaptation to cold is an anabolic process that involves enhanced *de novo* synthesis of lipids, proteins and organelles (2). Brown adipocytes engage protein quality control mechanisms to maintain cellular homeostasis under these stressful metabolic conditions (3) and we have demonstrated that NST requires an adaptive increase in proteasomal activity (4). The increase in transcription of proteasomal subunits was regulated by the ER-localized transcription factor nuclear factor erythroid 2–like 1 (Nfe2l1, also known as Nrf1 or TCF11). Interestingly, even in light of increased proteasomal protein degradation, cold adaptation resulted in enhanced protein ubiquitination (4), suggesting that the overall set point of the ubiquitin-proteasome system (UPS) is shifted in BAT after cold acclimatization. This probably also involves the machinery and regulators of ubiquitination itself.

The family of ubiquilin (*Ubqln*) proteins has recently emerged as versatile components of protein quality control. Ubiquilins are shuttle proteins that assist protein folding as chaperones, as well as facilitate degradation of ubiquitinated substrates through the UPS, endoplasmic reticulum-associated protein degradation (ERAD), and autophagy (5–7). To this date, five mammalian ubiquilins have been identified, 1, 2, 3, 4 and L, of which *Ubqln3* and *UbqlnL* are exclusively found in the testis (8, 9). *Ubqln1*, *2* and *4* are known to share a similar domain structure, which comprises a carboxy-terminal Ubiquitin-associated domain (UBA), heat-shock-chaperonin-binding motifs and an amino-terminal Ubiquitin-like domain (UBL). The UBA-UBL construct enables the interaction of ubiquilins with ubiquitinated proteins and the proteasome, which allows them to act as multifaceted adaptor molecules (10).

Based on the dynamic nature of UPS regulation in BAT during cold adaptation, it is likely that there are additional mechanisms supporting the function of Nfe2l1. In that vein, the role of ubiquilins in BAT has not been investigated so far. We hypothesized that Ubiquilins, with their function of targeting ubiquitinated proteins and facilitating their degradation by the UPS, could play a significant role in maintaining proteostasis in brown adipocytes. Here we investigate the role of ubiquilins for proteostasis and NST in immortalized and primary brown adipocytes.

## Material & Methods

### Mice

All animal experiments were performed according to procedures approved by the animal welfare committees of the government of Upper Bavaria, Germany (ROB-55.2-2532.Vet_02-20-32) and performed in compliance with German Animal Welfare Laws. Animals were housed in individually ventilated cages at room temperature (22°C) with a 12-h light–dark cycle. All mouse housing and husbandry occurred on standard chow diet (Ssniff). We used 12 weeks old C57BL/6J (Janvier) wild-type mice, which we housed at 30°C for 7 days for thermoneutral acclimatization. For cold exposure and cold adaptation, mice were housed at 4°C for 24 h and 1 week, respectively.

### Cell Culture and Treatments

We used an immortalized brown preadipocyte cell line (imBAT) (11), which we differentiated into mature brown adipocytes *in vitro*. The cells were induced at confluence for 2 days and differentiated for 2 days followed by 1 day of cultivation in standard medium [DMEM GlutaMax (Gibco), 10% fetal bovine serum (Sigma-Aldrich), 1% penicillin-streptomycin (Sigma-Aldrich)]. For induction the standard medium was supplemented with 850 nM human insulin (Sigma-Aldrich), 1 µM dexamethasone (Sigma-Aldrich, in 100% ethanol), 1 µM T3 (Sigma-Aldrich, in 1 M NaOH), 1 µM rosiglitazone (Cayman Chemicals, in 100% DMSO), 500 nM IBMX (Sigma-Aldrich, in 100% DMSO) and 125 nM indomethacin (Sigma-Aldrich, in 100% ethanol). The differentiation medium contained 1 µM T3 and 1 µM rosiglitazone. All treatments were performed on the 5^th^ day of differentiation. Proteasome inhibitors bortezomib (Selleckchem), epoxomicin (Millipore) and MG-132 (Calbiochem) were used at 100 nM in 100% DMSO and incubated for 6 h. For β3-adrenergic stimulation, cells were treated with 1 µM CL316,243 (Tocris, in distilled H_2_O) for 6 h.

### Primary Cell Preparation and Culture

For primary cell experiments, mature adipocytes were differentiated from preadipocytes, isolated from the stromal-vascular fraction of adipose tissue of 4 weeks old C57BL/6J (Janvier) wild-type mice. The mice were sacrificed by cervical dislocation, the respective adipose tissues were harvested and pooled. The tissues were minced, weighted and digested in DMEM/F-12 (Sigma-Aldrich), 1% PenStrep (Sigma-Aldrich), 15 mg/mL fatty acid free BSA (Sigma-Aldrich), 1 mg/mL collagenase type 2 (Worthington) and 0.1 mg/mL DNase 1 (Roche) for 30 – 45 min at 37°C. For BAT, the digestion mix was supplemented with 1.2 U/mL Dispase (Roche). The digestion was stopped by adding DMEM/F-12 (+10% FBS, +1% PenStrep) in a 1:5 ratio. The digest was filtered through a 100 µm strainer and centrifuged at room temperature for 10 min at 500 xg. The mature adipocyte and the supernatant were aspirated, the pellet suspended in DMEM/F-12 (+10% FBS, +1% PenStrep) and filtered through a 70 µm strainer. After another centrifugation step at room temperature for 10 min at 500 xg, the pellet was again suspended in DMEM/F-12 (+10% FBS, +1% PenStrep), filtered through a 30 µm strainer and eventually plated in T75 flasks. The medium was changed the next day, to remove debris from the digestion and then every other day until the cells reached confluency. To differentiate the preadipocytes into mature adipocytes, cells were induced for 2 days and differentiated for 2 days followed by 1 day cultivation in standard medium. The induction medium consisted of DMEM/F-12 (+10% FBS, +1% PenStrep) supplemented with 340 nM human insulin (Sigma-Aldrich), 1 µM dexamethasone (Sigma-Aldrich, in 100% ethanol), 1 µM T3 (Sigma-Aldrich, in 1 M NaOH), 1 µM rosiglitazone (Cayman Chemicals, in 100% DMSO), 500 nM IBMX (Sigma-Aldrich, in 100% DMSO). The differentiation medium for white adipocytes from gonadal white adipose tissue (GWAT) consisted of DMEM/F-12 (+10% FBS, +1% PenStrep) with 10 nM human insulin and 2 µM T3. For beige and brown adipocytes from subcutaneous adipose tissue (SCAT) and BAT, respectively, DMEM/F-12 (+10% FBS, +1% PenStrep) was supplemented with 10 nM human insulin, 2 µM T3 and 1 µM rosiglitazone.

### Reverse Transfection and RNAi

For RNA interference experiments, we reverse transfected imBAT and primary brown adipocytes with SMARTpool siRNA (Dharmacon) on the 3^rd^ day of differentiation. Lipofectamine™ RNAiMAX transfection reagent (Thermo Fisher Scientific) was used according to manufacturer’s instructions. SMARTpool siRNAs for *Nfe2l1*, *Ubqln1, 2* and *4* were used in a final concentration of 30 nM for single knockdowns. For double and triple knockdown of *Ubqln1*, *2* and *4* SMARTpool siRNAs were mixed in an equimolar ratio with final concentrations of 60 nM and 90 nM, respectively. After 24 h the transfection mix was replaced with the standard medium and the cells were incubated for another 24 h, after which we treated or directly harvested them.

### RNA Extraction, cDNA Synthesis and RT-qPCR

To extract total RNA from frozen adipose tissue and cells we used the NucleoSpin^®^ RNA kit (Macherey-Nagel) according to the manufacturer’s instructions. RNA concentrations were measured on a NanoDrop spectrophotometer (Thermo Fisher Scientific). To synthesize complementary DNA (cDNA), we reverse-transcribed 500 ng RNA with the Maxima™ H Master Mix 5x (Thermo Fisher Scientific) in a total volume of 10 μl. cDNA was diluted 1:40 with RNase-free H_2_O. Relative gene expression was quantified using quantitative real time-PCR. Each reaction contained 4 µL cDNA, 5 µL PowerUp™ SYBR Green Master Mix (Applied Biosystems) and 1 µL of 5 µM primer stock (full primer list in **Supplementary Table 1**). We used standard run conditions for Applied Biosystems SYBR Green Gene Expression Assays (2:00 min 50°C, 10:00 min 95°C, 40 cycles of 0:15 min 95°C, 1:00 min 60°C). Cycle thresholds (Cts) of genes of interest were normalized to *TATA-box binding protein (Tbp)* levels by the ΔΔCt-method and displayed as relative copies per *Tbp* or relative expression normalized to experimental control groups.

### Oil Red O Staining

To visualize and quantify lipid content in adipocytes, we used Oil Red O staining. Confluent cells were harvested 48 h after transfection, washed once with DPBS and incubated with zinc formalin solution (Merck) for 5 min at room temperature. Afterwards, zinc formalin was replaced with fresh zinc formalin and the cells were fixed for 48 h at 4°C. Adipocytes were washed with 100% isopropanol and dried at 37°C. Cells were stained with 0.5 ml 60% vol/vol Oil Red O solution (Sigma), 40% vol/vol distilled H_2_O for 1 h at room temperature and immediately washed 4x with distilled H_2_O afterwards. Pictures from each well were taken with a digital camera as well as with a microscope at 200x magnification before ORO stain was eluted with 100% isopropanol and transferred into a clear 96-well plate. To quantify the staining, absorption of the eluates was measured at 500 nm on a Tecan plate reader. Absorption was normalized by subtracting the absorbance of 100% isopropanol and displayed as fold change to *Scramble.*

### Extracellular Flux Analysis (Seahorse)

For extracellular flux analysis, we reverse transfected imBAT and plated them at 25,000 cells per well in a 24-well Seahorse plate in two independent experiments. To ensure even distribution, cells were seeded in 50 µl of medium and kept for 2 h at room temperature. Then, 175 µl medium were added, and the cells were allowed to fully attach at 37°C overnight. On the day before the experiment, Seahorse cartridges with the sensors were equilibrated in XF calibrant solution (Agilent) at 37°C in a non-CO2 chamber overnight. The experiment was performed on the 5th day of differentiation. Cells were treated with 100 nM bortezomib for 6 h or the corresponding amount of DMSO (Sigma-Aldrich) as a control treatment. XF DMEM pH 7.4 medium (Agilent) was supplemented fresh on the day of the experiment with 10 mM glucose, 1 mM pyruvate and 2 mM L-glutamine (Seahorse medium, all reagents from Agilent). Cells were carefully washed 2x with 1 ml Seahorse medium before incubating them with 500 µl of Seahorse medium for approximately 45 min at 37°C without CO_2_. During the measurement norepinephrine (Sigma-Aldrich), oligomycin (Sigma-Aldrich), FCCP (Sigma-Aldrich) and rotenone–antimycin A (Sigma-Aldrich) were injected *via* the port of the Seahorse cartridge. Final concentrations were 1 µM norepinephrine (in 100% H_2_O), 1 µM oligomycin, 4 µM FCCP and 0.5 µM rotenone–antimycin A (all in 100% DMSO). The protocol was 3 min mix, 2 min wait and 3 min measure for each time point. For normalization of respiration to live cell count, the cell plate was retrieved after the run, and we quantified the cell count using the CyQUANT^®^ Cell Proliferation Assay Kit (Thermo Fisher Scientific) according to manufacturer’s instructions.

### Protein Extraction and Western Blotting

We lysed cells in RIPA buffer [150 mM NaCl (Merck), 5 mM EDTA (Merck), 50 mM Tris pH 8 (Merck), 0.1% wt/vol SDS (Carl Roth), 1% wt/vol IGEPAL^®^ CA-630 (Sigma-Aldrich), 0.5% wt/vol sodium deoxycholate (Sigma-Aldrich)] freshly supplemented with protease inhibitors (Sigma-Aldrich) in a 1:100 ratio. The lysate was centrifuged at 4°C for 30 min at 21,000xg, to clear the lysate from debris and lipids. Protein concentrations were determined using the Pierce BCA Protein Assay (Thermo Fisher Scientific) according to the manufacturer’s instructions. For western blotting, lysates were adjusted to a final concentration of 0.85 µg/µl in 1x Bolt™ LDS Sample buffer (Thermo Fisher Scientific) supplemented with 5% vol/vol 2-mercaptoethanol (Sigma-Aldrich). For SDS-PAGE we used Bolt™ 4–12% Bis-Tris gels (Thermo Fisher Scientific) with Bolt™ MOPS SDS running buffer. After separation, proteins were transferred onto a 0.2 µm PVDF membrane (Bio-Rad) using the Trans-Blot^®^ Turbo™ system (Bio-Rad) at 12 V, 1.4 A for 16 min. The membrane was briefly stained with Ponceau S (Sigma-Aldrich) to verify successful transfer and blocked in 1x Roti-Block (Carl Roth) for 1 h at room temperature. Primary antibodies (Cell signaling) were used in a 1:1,000 ratio in 1x Roti-Block overnight at 4°C. After washing 4× for 10 min with TBS-T (200 mM Tris (Merck), 1.36 mM NaCl (Merck), 0.1% vol/vol Tween 20 (Sigma)), secondary antibodies (Santa Cruz) were applied in a 1:10,000 ratio in Roti-Block for 1 h at room temperature. Then membranes were washed again 3× for 10 min in TBS-T and developed using SuperSignal West Pico PLUS Chemiluminescent Substrate (Thermo Fisher Scientific) and a Chemidoc imager (Bio-Rad). We analyzed the digital images with the Image Lab software (Bio-Rad). Uncropped images can be found in (**Supplementary Figure 1**).

### Proteasomal Activity Assay

For the proteasomal activity assay, we lysed cells with a buffer containing 40 mM TRIS pH 7.2 (Merck), 50 mM NaCl (Merck), 5 mM MgCl_2_(hexahydrate) (Merck) and 10% vol/vol glycerol (Sigma-Aldrich) freshly supplemented with 2 mM β-mercaptoethanol (Sigma-Aldrich) and 2 mM ATP (Sigma-Aldrich). To measure chemotrypsin-like, trypsin-like and caspase-like activity, we used the Proteasome Activity Fluorometric Assay (UBP Bio) according to the manufacturer’s instructions. For the normalization of proteasomal activity to total protein content, we used the Bio-Rad Protein Assay Kit II to determine protein concentration in our samples.

### Statistics

Data were expressed as the mean ± standard error of the mean (SEM). For comparing three and more groups we used one-way ANOVA with Bonferroni post-hoc test as indicated in the figure legends. For comparing two variables we used two-way ANOVA with Bonferroni post-hoc test. Analysis was performed using R, Microsoft Excel and/or GraphPad Prism. P <0.05 was considered significant, as indicated by asterisks and letters in the figures legends.

## Results

### *Ubqln1* Is Expressed in Adipose Tissue and Induced by Cold and Proteasome Inhibition

**Figure 1:**
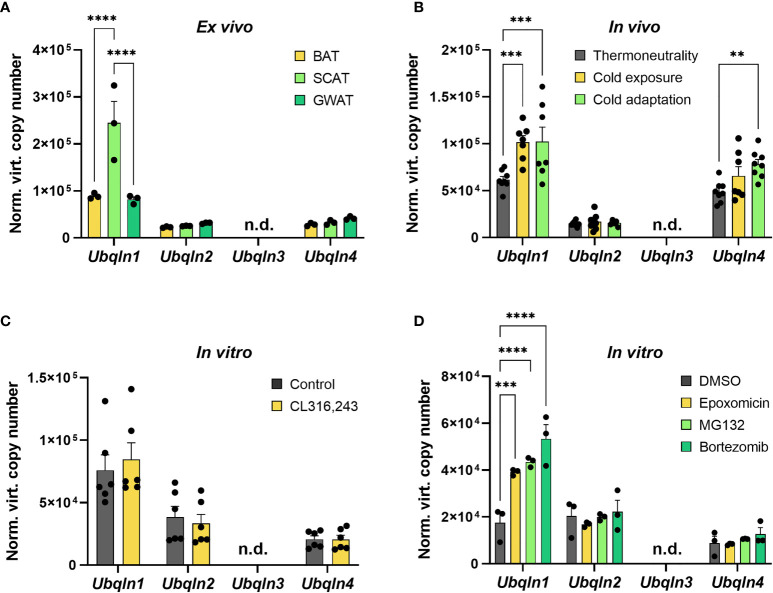
*Ubqln1* is highly expressed in adipose tissue and induced by cold & proteasome inhibition. Absolute mRNA expression calculated from Ct values of *Ubqln1*, *Ubqln2, Ubqln3* & *Ubqln4* in **(A)** Primary adipocytes from BAT, SCAT & GWAT (*n* = 3 technical replicates). **(B)**, BAT from mice kept at 30°C for 7 days (thermoneutrality), 4°C for 24 h (cold exposure) and 4°C for 7 days (cold adaptation) (*n* = 8 biological replicates). **(C)** imBAT untreated or treated with 1 µM CL316,243 for 6 h (n = 6 technical replicates from 2 independent experiments). **(D),** imBAT treated with DMSO (control), 100 nM epoxomicin, 100 nM MG132 or 100 nM bortezomib for 6 h (n = 6 technical replicates from 2 independent experiments). Throughout, data are mean ± SEM. *P_adj_ < 0.05, **P_adj_ < 0.01, ***P_adj_ < 0.001, ****P_adj_ < 0.0001 by two-way ANOVA with Bonferroni *post-hoc* test **(A–D)**. n.d., not detected; SCAT, subcutaneous white adipose tissue; GWAT, gonadal white adipose tissue.

As nothing is known about ubiquilins in BAT, we first investigated their gene expression levels in primary and immortalized adipocytes as well as in BAT ex vivo and second how these are impacted by cold and proteasome inhibition. In primary cells from intrascapular brown, inguinal subcutaneous and gonadal white adipose tissue (WAT), *Ubqln1, 2* and *4* expression was found to be expressed whereas *Ubqln3* was undetectable (**Figure 1A**). *Ubqln2* and *Ubqln4* were expressed at similar levels in all three adipocyte types. Interestingly, *Ubqln1* was approximately higher expressed compared to *Ubqln2* and *4* with highest expression in adipocytes from the subcutaneous WAT. In BAT from 12-week-old mice, which were kept at 4°C for 24 h (cold exposure) and 7 days (cold adaptation), we found that *Ubqln1* and *Ubqln4* expression was higher compared to mice kept at 30°C (thermoneutrality) (**Figure 1B**). We confirmed that the cold challenge was successful by increased expression of thermogenic markers *Ucp1* and *Ppargc1a* (**Supplementary Figures 2A, B**). Compared to primary brown adipocytes, we found a similar expression pattern in imBAT, which remained unchanged upon stimulation with beta3-agonist CL316,243 (**Figure 1C**). Proteasome inhibition with epoxomicin, MG132 or bortezomib respectively, resulted in higher *Ubqln1* expression compared to DMSO treated controls (**Figure 1D**). We were unable to detect *Ubqln3* mRNA in any of our samples, which is in line with a previous report that *Ubqln3* is specific to testis (9). Thus, *Ubqln1, 2* and *4* are expressed in adipose tissue, yet only *Ubqln1* is positively regulated by cold and proteasome inhibition.

### Silencing of *Ubqln1*, *2* and *4* Does Not Cause ER Stress or Inflammation

**Figure 2:**
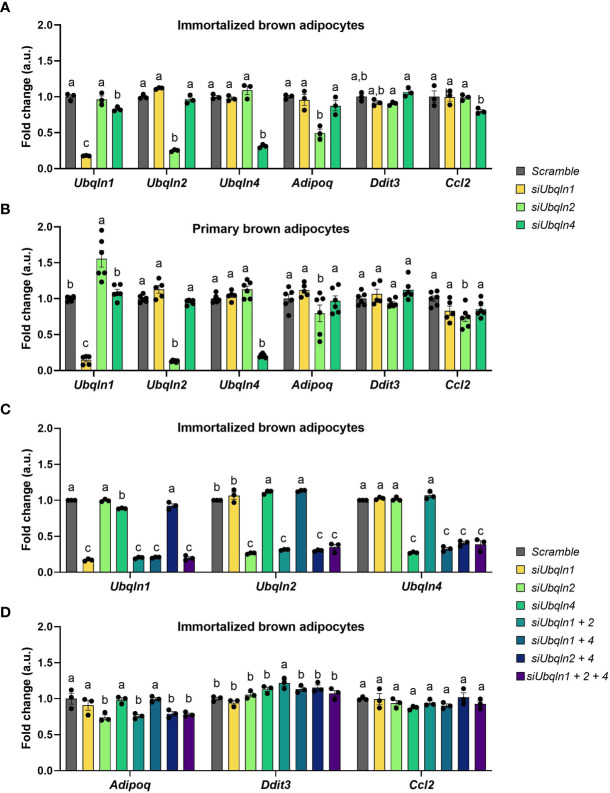
Silencing of *Ubqln1, 2 and 4* in brown adipocytes does not cause ER stress or inflammation. Ubiquilins were silenced by siRNA-mediated knockdown in imBAT and primary brown adipocytes. Relative gene expression measured by qPCR in: **(A)**, imBAT (*n* = 3 technical replicates). **(B)**, primary brown adipocytes (*n* = 5-6 technical replicates from 2 independent experiments). **(C, D),** imBAT (n = 3 technical replicates). Throughout, data are mean ± SEM. Statistical testing was done by two-way ANOVA with Bonferroni post-hoc test. Different small letters indicate differences in mean compared to *Scramble* with at least P_adj_ < 0.05.

Next, we determined the significance of ubiquilins in brown adipocyte homeostasis. We used RNA interference to silence the gene expression of *Ubqln1, 2* and *4* in immortalized and primary adipocytes to investigate their functional relevance. We explored established surrogate markers of stress responses and adipocyte function linked to BAT proteostasis (4). While our RNAi strategy was efficient, knockdown of any of the *Ubqln* genes did not result in changes in the mRNA expression of surrogate stress markers, *Ddit3* and *Ccl2* (**Figures 2A, B**). Knockdown of *Ubqln2* resulted in lower *Adipoq* expression in both immortalized and primary brown adipocytes, which suggests that adipocyte health is affected under these conditions. Since *Ubqln1, 2* and *4* share the same domain structure and their sequences are highly similar, we hypothesized that they could compensate for each other (12). This was also supported by upregulation of *Ubqln2* after knockdown of *Ubqln1* in primary brown adipocytes (**Figure 2B**). To circumvent this effect, we studied all possible combinations of *Ubqln1, 2* and *4* siRNAs in single, double and triple *Ubqln* knockdown experiments in imBAT (**Figure 2C**). This resulted in lower *Adipoq* expression in conditions with *Ubqln2* knockdown compared to the control cells (**Figure 2D**). *Ddit3* and *Ccl2* expression remained largely unaffected. Hence, loss of *Ubqln1, 2* or *4* did not increase ER stress or inflammation marker genes, alone or in combination.

### Silencing of Ubiquilins Does Not Impair Adipocyte Health and Non-Shivering Thermogenesis

**Figure 3:**
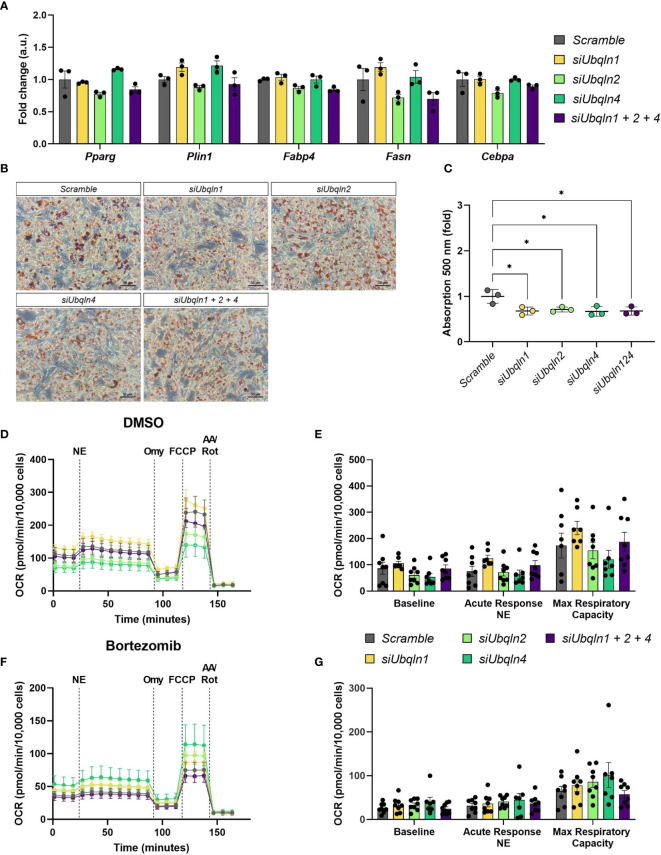
Silencing of *Ubqln1, 2 and 4* lowers lipid content but does not impair adipocyte health. Ubiquilins were silenced by siRNA-mediated knockdown in imBAT. **(A)**, Relative gene expression measured by qPCR (*n* = 3 technical replicates mean ± SEM). **(B, C)**, Oil red O staining and quantification. **(D, E),** Oxygen consumption rate (OCR) of imBAT treated with DMSO (control) or **(F, G)**, 100 nM bortezomib for 6 h. Data are mean ± SEM. (*n* = 7-8 technical replicates of 2 independent experiments). *P_adj_<0.05, **P_adj_ < 0.01, ***P_adj_ < 0.001, ****P_adj_ < 0.0001 by two-way ANOVA *versus Scramble* with Bonferroni post-hoc test **(A, C, E, G)**.

As we observed a reduction in *Adipoq* mRNA levels in both immortalized and primary brown adipocytes, we next investigated how loss of ubiquilins impacts adipocyte health. To our surprise, the expression levels of other adipocyte marker genes such as *Pparg*, *Plin1*, *Fabp4*, *Fasn* and *Cebpa* remained unchanged by *Ubqln* single or triple knockdown (**Figure 3A**. However, compared to the control cells we observed a lower lipid content in imBAT (**Figures 3B, C** and **Supplementary Figure 3**). Next, we investigated if loss of ubiquilins impacts NST. We analyzed norepinephrine-induced respiration and mitochondrial stress tests as the gold standard of measuring NST in cells. Neither single nor triple knockdown affected basal cellular respiration, the response to norepinephrine stimulation or maximal respiratory capacity (**Figures 3D, E**). To investigate if ubiquilins participate in the coordinated cellular response to proteasome inhibition, which has been shown to impair mitochondrial function in brown adipocytes (4), we treated imBAT cells with the compound bortezomib. While pretreatment with bortezomib led to lower levels of respiration, this response was independent of single or triple knockdown of ubiquilins (**Figures 3F, G**).

### Ubiquilins Are Dispensable for Proteostasis in Brown Adipocytes

**Figure 4:**
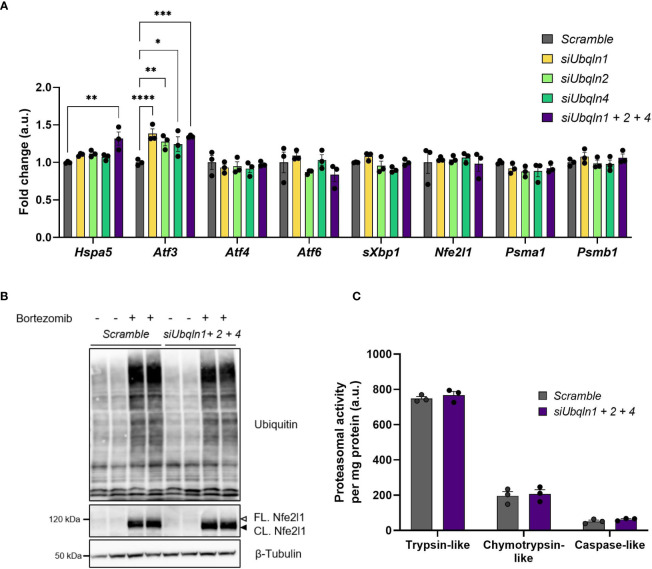
*Ubqln1, 2 and 4* are not required for maintaining proteostasis in brown adipocytes. Ubiquilins were silenced by siRNA-mediated knockdown in imBAT. **(A)**, Relative gene expression measured by qPCR (*n* = 3 technical replicates mean ± SEM). *P_adj_ < 0.05, **P_adj_ < 0.01 and ***P_adj_ < 0.001 by one-way ANOVA with Bonferroni post-hoc test. **(B),** Representative ubiquitin immunoblot, imBAT treated with DMSO (control) or 100 nM bortezomib for 6 h (*n* = 2 technical replicates, representative blot of 2 independent experiments). **(C)**, Proteasomal activity (*n* = 3 technical replicates, mean ± SEM). *P_adj_ < 0.05, ***P_adj_ < 0.001 by one-way **(A)** or two-way **(D)** ANOVA *versus Scramble* with Bonferroni post-hoc test **(A, C)**.

As we found that *Ubqln1* expression in brown adipocytes is strongly induced by proteasome inhibition, we hypothesized that Ubiquilins are involved in maintaining proteostasis. Indeed, knockdown of *Ubqln1, 2* and *4* resulted in higher *Atf3* gene expression, albeit the effect was small but significant (**Figure 4A**). Other stress markers related to accumulation of misfolded proteins or ER stress such as *Atf4*, *Atf6* and s*Xbp1* were unchanged and *Hspa5* was only higher in the triple knockdown condition. Another important player for brown fat proteostasis is the transcription factor *Nfe2l1*, which activates the transcription of proteasomal subunits in a bounce-back-mechanism in response to proteasomal inhibition and cold (4). It is conceivable that *Nfe2l1* counteracts accumulation of misfolded proteins and ER stress as a consequence of diminished *Ubqln* expression through increased proteasome formation. However, we neither observed differences in *Nfe2l1* expression nor in the expression of two of its downstream targets *Psma1* and *Psmb1*. We also tested whether, the induction of *Atf3* in response to *Ubqln* knockdown was mediated by *Nfe2l1*, but *Atf3* expression was unchanged by *Ubqln* triple knockdown in combination with Nfe2l1 knockdown (**Supplementary Figure 4**). In line with these findings, single *Ubqln* knockdown (**Supplementary Figure 5**) or triple *Ubqln* knockdown neither affected the levels of ubiquitinated proteins nor Nfe2l1 protein levels, neither under basal conditions nor after proteasome inhibition (**Figure 4B**). Triple knockdown did also not result in lower proteasomal activity (**Figure 4C**). Thus, ubiquilins are dispensable to maintain proteostasis in brown adipocytes under basal and proteasome inhibited conditions and their absence is not linked to Nfe2l1 activation.

## Discussion

The adaptation to sustained cold is a challenging process for thermogenic adipocytes. These cells undergo extensive remodeling for enhanced and sustainable oxidative metabolism, which involves the synthesis of new proteins, lipids and entire organelles (2). We have previously shown that this process requires enhanced proteasomal protein quality control, transcriptionally mediated by Nfe2l1 (4). However, even with increased proteasomal degradation, cold adaptation still results in increased protein ubiquitination (4). The mechanisms dynamically coupling ubiquitination of proteins to proteasomal degradation in brown adipocytes remain elusive. Here, we investigated the role of ubiquilins for proteostasis and NST in brown adipocytes.

Ubiquilins are multifaceted shuttling proteins, which have an amino-terminal UBL and a carboxy-terminal UBA domain, which enable them to interact with both mono- and poly-ubiquitinated cargo as well as facilitate their degradation through the UPS. This role seems to be their predominant function, as it is conserved from yeast to humans (10). We show that *Ubqln1, 2* and *4* are highly expressed in BAT and that their expression, most notably of *Ubqln1* is induced by cold and proteasomal inhibition. *Ubqln1*, *2* and *4* are highly homologous and share a similar domain structure. It is therefore likely that they share similar functions and might compensate for the loss of each other (13). However, silencing of one or the combination of ubiquilins by RNAi did neither impact ubiquitin levels nor proteasomal activity under basal conditions or after treatment with proteasome inhibitors. Consequently, except for mildly upregulated expression of *Atf3*, a stress marker in response to various physiological stressors, no changes in ER stress or inflammation were observed under these conditions.

We could speculate that *Nfe2l1* as the major regulator of proteostasis in brown adipocytes, is involved in upregulation of *Atf3* upon knockdown of ubiquilins. However, we did not investigate this in an epistasis-type of experiment, since we did not find any changes in Nfe2l1 gene and protein expression. Another limitation of this study is that we only silenced *Ubqln1*, *2* and *4* by RNAi. Presumably, knockout of one or all ubiquilins would have a stronger effect on proteostasis and NST. In mice, transgenic knockout of *Ubqln1* in neurons aggravated brain injury and delayed functional recovery after ischemic stroke. Ubiquitous overexpression of *Ubqln1*, however, resulted in reduced neuronal damage (14). Similarly, cardiomyocyte-specific knockout of *Ubqln1* in a mouse model of myocardial ischemia-reperfusion injury resulted in enlarged infarct size and late-onset cardiomyopathy, whereas overexpression of *Ubqln1* resulted in reduced infarct size (15). Moreover, in humans, mutations in *Ubqln1, 2* and *4* are strongly associated with the onset of neurodegenerative diseases such as dementia, Alzheimer’s disease and amyotrophic lateral sclerosis (12, 16–18). These data strongly suggest that impairment or loss of function of ubiquilins are associated with numerous pathologies in both humans and rodents.

In Drosophila loss of Ubqn, the homologue of mammalian ubiquilins, results in ER expansion and activation of the PKR-like ER kinase (PERK) pathway, one branch of the unfolded protein response (5). According to our original hypothesis that ubiquilins might be involved in ubiquitination of proteins and their proteasomal degradation during NST, we measured norepinephrine-induced respiration but NST was intact despite the loss of ubiquilins. However, we found reduced lipid content compared to control cells. Lipogenesis and lipid droplet biosynthesis takes place in the ER and ER stress, even if driven by proteotoxic stressors disrupt lipid homeostasis (3). In light of the absence of overt ER stress upon loss of ubiquilins, the changes in lipid content point to a more specific role of ubiquilins in lipid metabolism beyond proteostasis. While this warrants further investigation, we did not observe any functional change in NST.

Yeast has only one ubiquilin isoform Dsk2, but other UBL/UBA proteins such as Rad23. None of these individual proteins are essential in yeast, but combined loss of Rad23, Dsk2 and the proteasome ubiquitin receptor Rpn10/S5a results in mitotic arrest (19). This suggests that, at least in yeast, UBL/UBA proteins are redundant and may have overlapping substrate specificity. In future, it will be important finding those UBL/UBA proteins that participate in ubiquitination in brown adipocytes. In summary, in this study, we investigated the role of ubiquilins for brown adipocyte proteostasis and thermogenesis. We found that despite high expression levels of *Ubqln 1, 2* and *4*, these ubiquilins are only minor regulators of brown adipocyte function.

## Data Availability Statement

The raw data supporting the conclusions of this article will be made available by the authors, without undue reservation.

## Ethics Statement

The animal study was reviewed and approved by Government of Upper Bavaria, Germany (ROB-55.2-2532.Vet_02-20-32).

## Author Contributions

CM performed experiments and analyzed data. SK provided samples and assisted with experiments. CM and AB conceptually designed the study, interpreted the data and wrote the manuscript. All authors contributed to the article and approved the submitted version.

## Funding

AB and this work was supported by the Deutsche Forschungsgemeinschaft Sonderforschungsbereich 1123 (B10), the Deutsches Zentrum für Herz-Kreislauf-Forschung Junior Research Group Grant, and the European Research Council Starting Grant Proteofit.

## Conflict of Interest

The authors declare that the research was conducted in the absence of any commercial or financial relationships that could be construed as a potential conflict of interest.

## Publisher’s Note

All claims expressed in this article are solely those of the authors and do not necessarily represent those of their affiliated organizations, or those of the publisher, the editors and the reviewers. Any product that may be evaluated in this article, or claim that may be made by its manufacturer, is not guaranteed or endorsed by the publisher.

## Abbreviations

BAT, Brown adipose tissue; GWAT, Gonadal white adipose tissue; imBAT, Immortalized brown pre-adipocyte cell line; NST, Non-shivering thermogenesis; SCAT, Subcutaneous adipose tissue; UBA, Ubiquitin-associated domain; UBL, Ubiquitin-like domain; WAT, White adipose tissue.
