# Supplementary material for: Role of Ubiquilins for Brown Adipocyte Proteostasis and Thermogenesis
Source: Front Endocrinol (Lausanne). 2021 Sep 28;12:739021. doi: 10.3389/fendo.2021.739021 (PMC8505974; doi:10.3389/fendo.2021.739021)
Supplement: Supplementary file 1 [file DataSheet_1.docx]

Supplementary Material

**Supplementary Table 1 Primer list**

| Gene | Sequence forward primer | Sequence reverse primer |
| --- | --- | --- |
| *Adipoq* | ggagagaaaggagatgcaggt | ctttcctgccaggggttc |
| *Atf3* | gaggattttgctaacctgacacc | ttgacggtaactgactccagc |
| *Atf4* | ccttcgaccagtcgggtttg | ctgtcccggaaaaggcatcc |
| *Atf6* | ggacgaggtggtgtcagag | gacagctcttcgctttggac |
| *Ccl2* | ttaaaaacctggatcggaaccaa | gcattagcttcagatttacgggt |
| *Cebpa* | aaacaacgcaacgtggaga | gcggtcattgtcactggtc |
| *Ddit3* | ctggaagcctggtatgaggat | cagggtcaagagtagtgaaggt |
| *Fabp4* | ggatggaaagtcgaccacaa | tggaagtcacgcctttcata |
| *Fasn* | ccaaatccaacatgggaca | tgctccagggataacagca |
| *Hspa5* | tcatcggacgcacttggaa | caaccaccttgaatggcaaga |
| *Nfe2l1* | gacaagatcatcaacctgcctgtag | gctcacttcctccggtcctttg |
| *Plin* | caagcacctctgacaaggttc | gttggcggcatattctgctg |
| *Pparg* | tcgctgatgcactgcctatg | gagaggtccacagagctgatt |
| *Ppargc1a* | ttcatctgagtatggagtcgct | gggggtgaaaccacttttgtaa |
| *Psma1* | tgcgtgcgtttttgattttagac | ccctcagggcaggattcatc |
| *Psmb1* | cgttgaaggcataaggcgaaaa | ttccactgctgcttaccgag |
| *Tbp* | agaacaatccagactagcagca | gggaacttcacatcacagctc |
| *Ubqln1* | tgctgaataatcccctatttgc | tgtcggggttctgcattt |
| *Ubqln2* | gccacctactgaacaaccca | agccaggtcttgatttcgca |
| *Ubqln3* | gagccctgcctaagaggaa | tctccacttttggccatgat |
| *Ubqln4* | caatgcccagcaacaactc | gaaatctcacttctggcatcg |
| *Ucp1* | aggcttccagtaccattaggt | ctgagtgaggcaaagctgattt |
| *sXbp1* | ggtctgctgagtccgcagcagg | aggcttggtgtatacatgg |


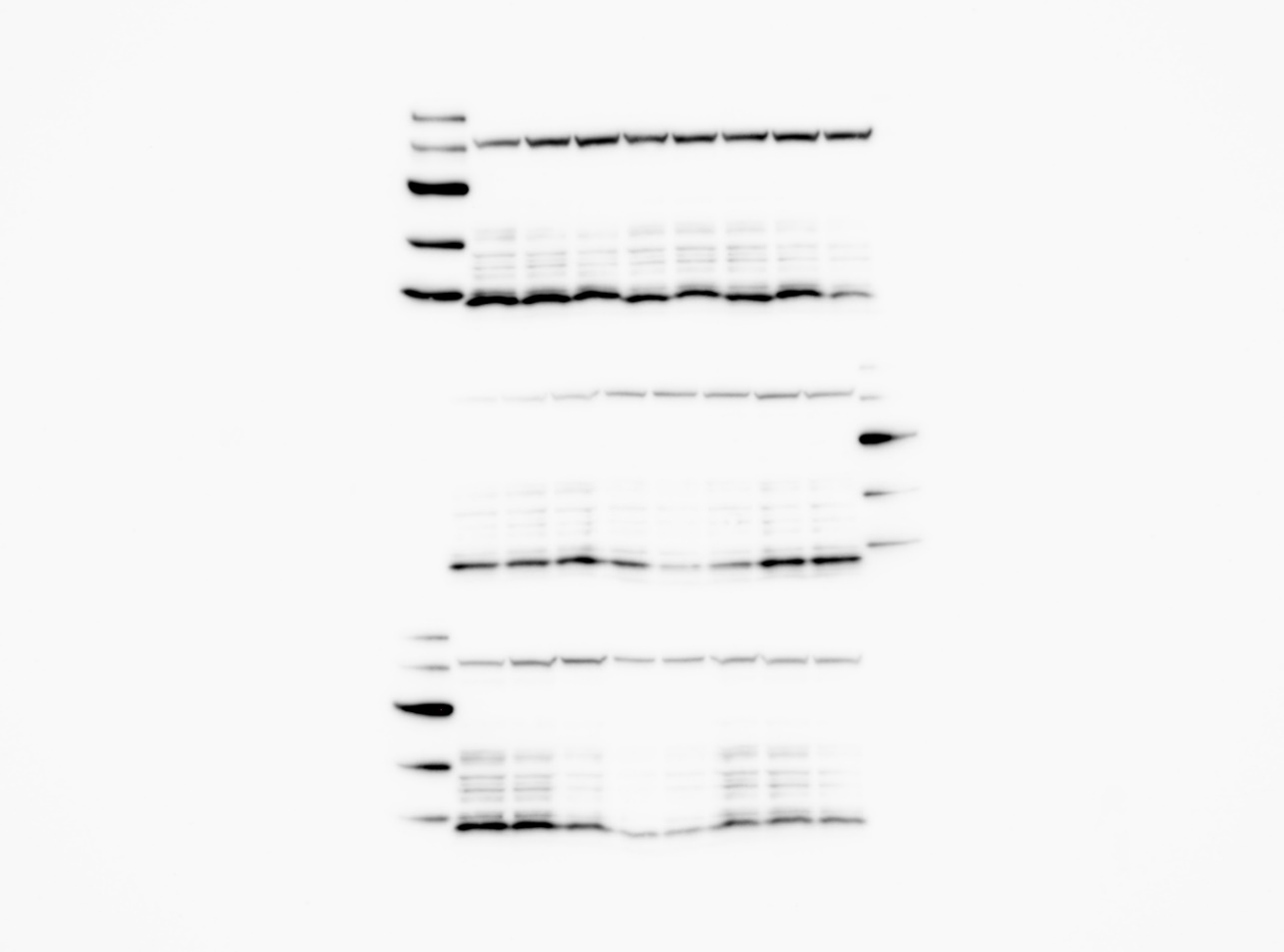

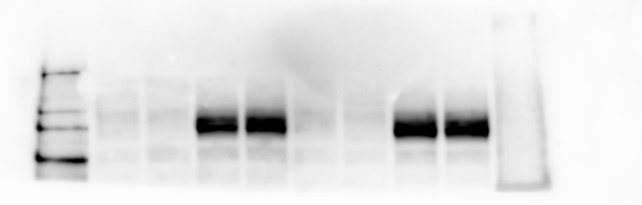

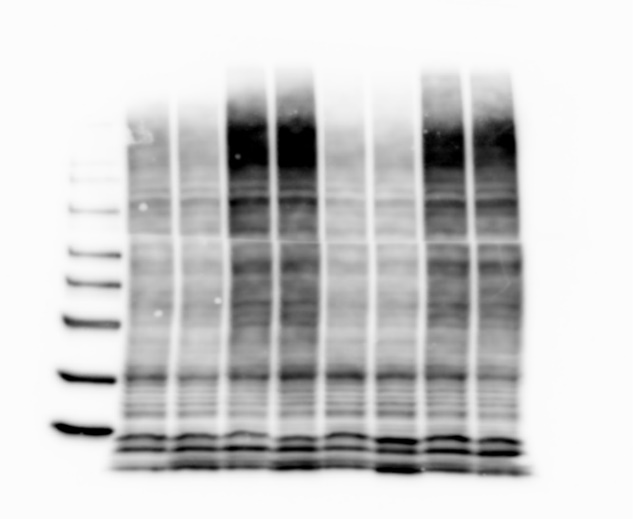


**A**

**B**

**C**

**Supplementary Figure 1: Uncropped Western blots.** (**A**) Anti-Ubiquitin (P4D1), cell signaling #3936, 1:1,000 dilution (B) Nfe2l1: Anti-TCF11/NRF1 (D5B10), cell signaling #8052, 1:1,000 dilution, (**C**) Anti-beta-Tubulin, cell signaling #2146, 1:1,000 dilution

**Supplementary Figure 2: Expression of thermogenic marker genes.** (**A-B**) BAT from mice kept at 30°C for 7 days (thermoneutrality), 4°C for 24 h (cold exposure) and 4 °C for 7 days (cold adaptation) (*n* = 8 biological replicates). (**C**) Primary brown adipocytes (*n* = 5-6 technical replicates from 2 independent experiments). Data are mean ± SEM. P_adj_<0.05, **P_adj_<0.01, ***P_adj_<0.001 by one-way ANOVA with Bonferroni post-hoc test.


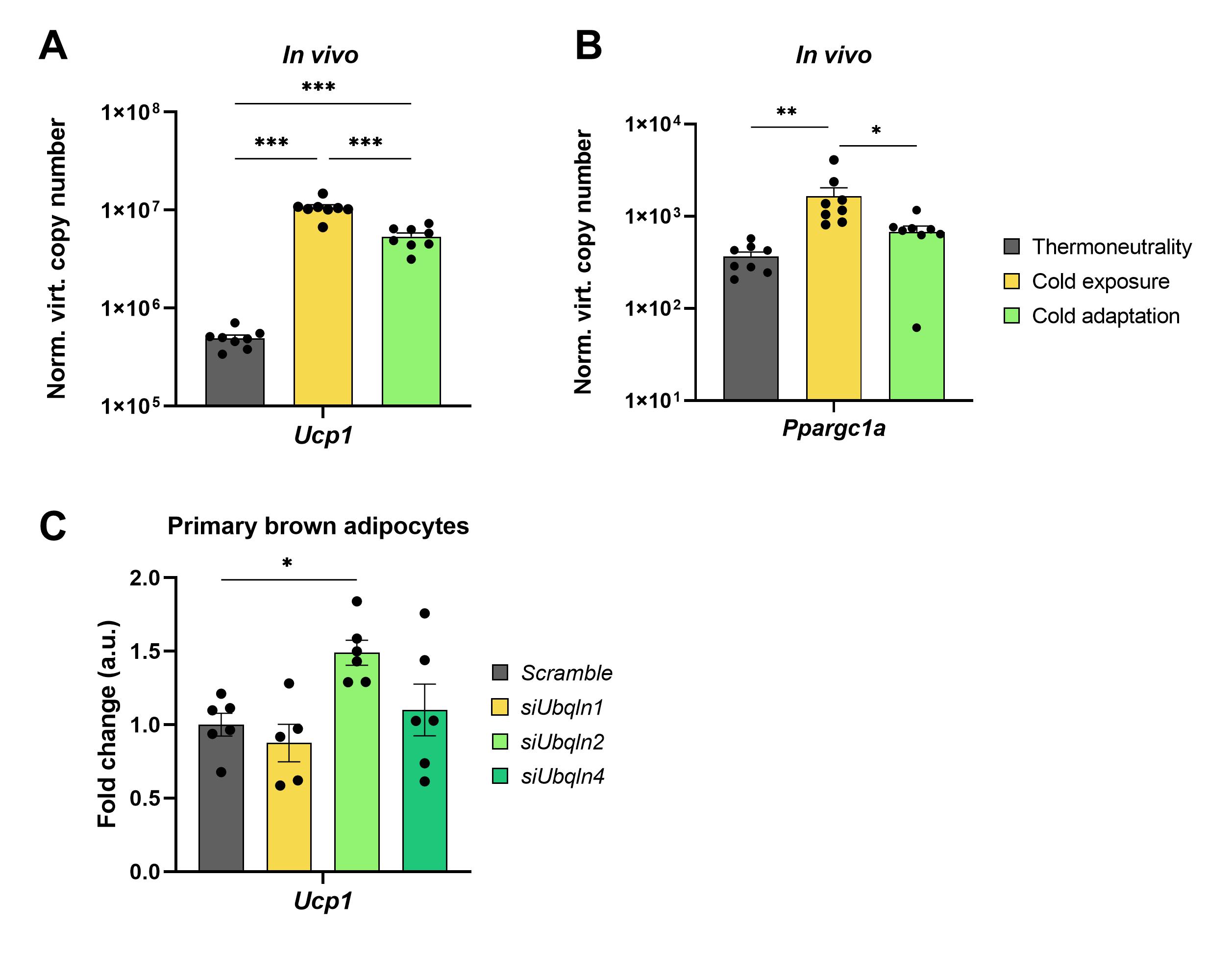

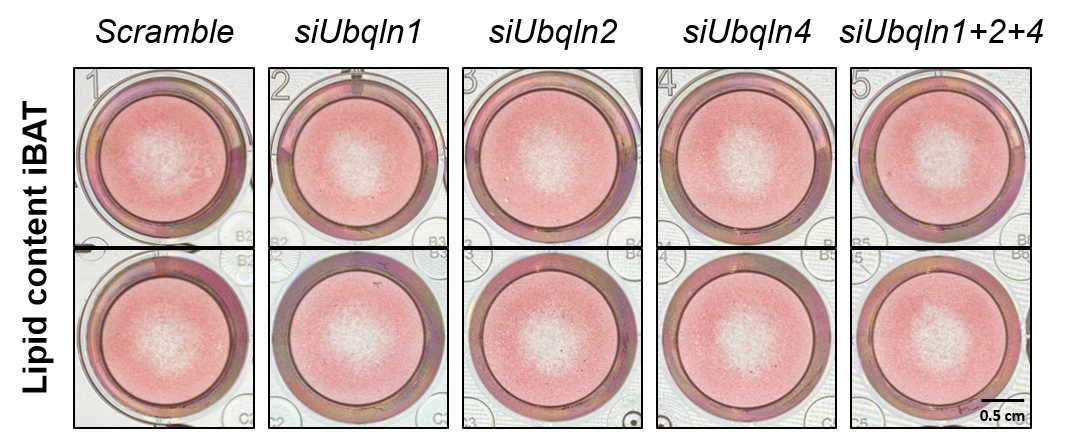


**Supplementary Figure 3 Oil red O staining of imBAT.**


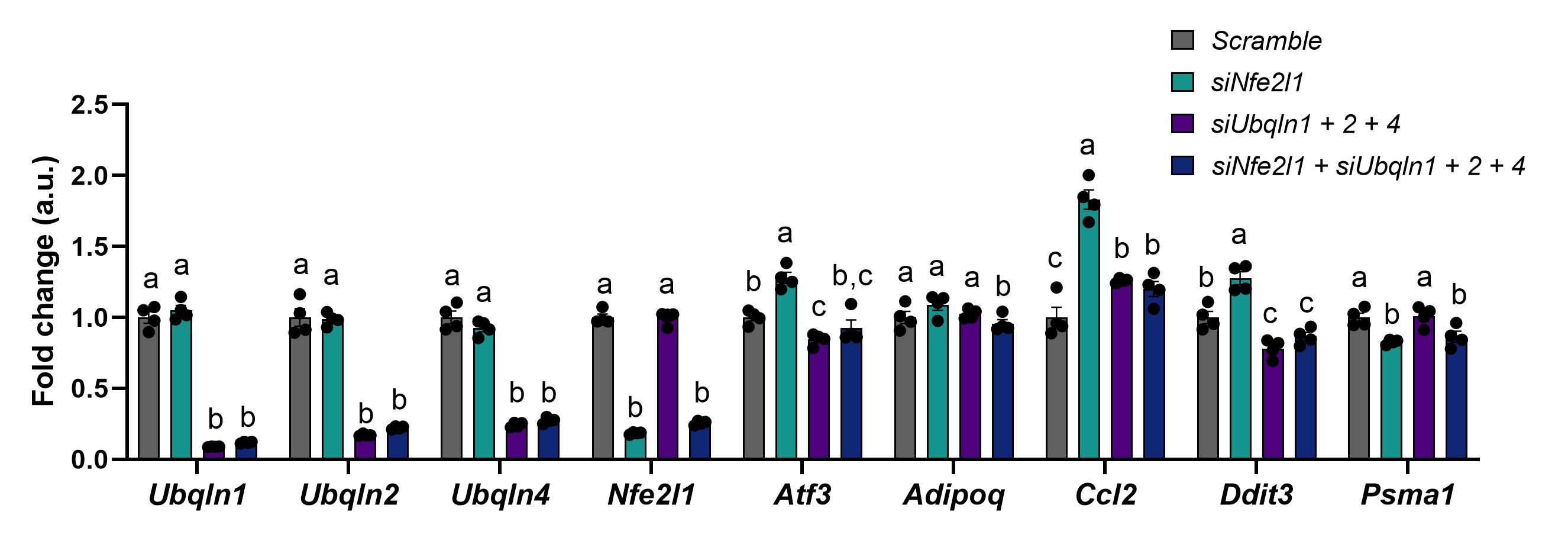


**Supplementary Figure 4: Induction of *Atf3* after ubiquilin knockdown is not mediated by Nfe2l1.** Ubiquilins and Nfe2l1 were silenced by siRNA-mediated knockdown in imBAT. Data are mean ± SEM (*n* = 4 technical replicates). Statistical testing was done by two-way ANOVA with Bonferroni post-hoc test.  Different small letters indicate differences in mean compared to *Scramble* with at least P_adj_<0.05.

**
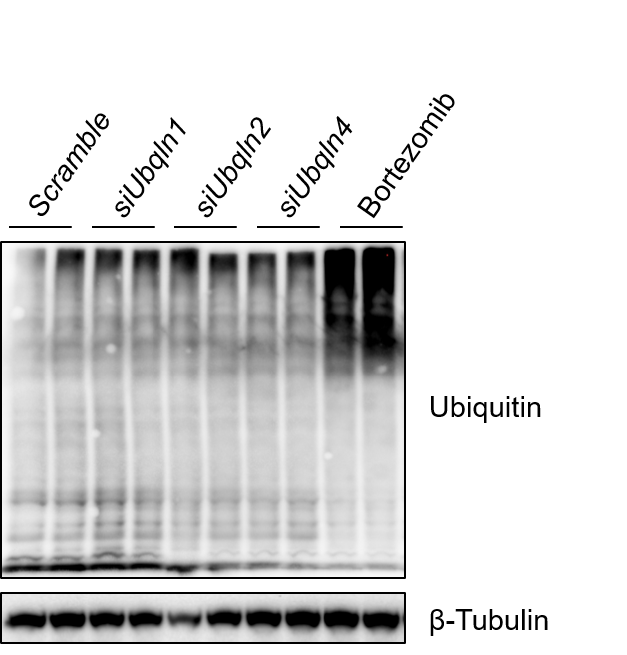
**

**Supplementary Figure 5: Global ubiquitin level after ubiquilin knockdown.** Representative ubiquitin immunoblot. Ubiquilins were silenced by siRNA-mediated knockdown in imBAT. Untransfected cells treated with 100 nM bortezomib for 6 h were used as a positive control for ubiquitination (*n* = 2 technical replicates, representative blot of 2 independent experiments).
